# Whole-body diffusion-weighted MRI versus CT for detection, restaging and operability assessment of recurrent ovarian carcinoma

**DOI:** 10.1186/1470-7330-14-S1-S7

**Published:** 2014-10-09

**Authors:** K Michielsen, I Vergote, K Op de beeck, F Amant, K Leunen, S Dymarkowski, P Moerman, F De Keyzer, V Vandecaveye

**Affiliations:** 1 Department of Radiology, Leuven Cancer Institute, University Hospitals Leuven, Leuven, Belgium; 2 Department of Obstetrics and Gynaecology, Leuven Cancer Institute, University Hospitals Leuven, Leuven, Belgium; 3 Department of Pathology, Leuven Cancer Institute, University Hospitals Leuven, Leuven, Belgium

## Aim

To evaluate whole body diffusion-weighted MR imaging (WB-DWI MRI) for detection, staging and operability assessment in recurrent ovarian cancer compared with CT.

## Methods

Fifty-one women suspected for recurrent ovarian cancer underwent 3-Tesla WB-DWI/MRI using 2 b-values (b=0-1000 s/mm²), T2- and contrast T1-weighted sequences in addition to CT. WB-DWI/MRI and CT were compared for per-patient detection of recurrence, per-site detection of disease extent including peritoneal, serosal, retroperitoneal, periportal and distant metastases and for detecting disease extent according to institutional operability criteria. Imaging findings were correlated with surgical/pathological findings or imaging follow-up for at least 6 months.

## Results

According to the reference standard, recurrence was confirmed in 48/51 patients. WB-DWI MRI showed 94% accuracy for detecting recurrence, versus 78% for CT. Per-site analysis showed significantly higher sensitivity of WB-DWI MRI over CT for assessing disease extent of the peritoneum, small bowel and colon mesentery and serosa (p<0.000001, p<0.000001 and p=0.00002, respectively), retroperitoneal suprarenal lymphadenopathies and periportal lesions (both p=0.031). Following institutional operability criteria, WB-DWI/MRI showed better sensitivity for detection of disease extent compromising operability; mesenteric root infiltration (p=0.008), carcinomatosis of small bowel (p=0.002) and colon (p=0.016), high volumetric peritoneal disease load (p=0.004) and irresectable distant metastases (p=0.016). WB-DWI MRI correctly predicted complete cytoreduction in 93% patients undergoing cytoreductive surgery versus 40% for CT.

## Conclusion

WB-DWI MRI showed higher accuracy compared with CT for recurrence detection while improving the sensitivity for staging and operability assessment of disease extent. WB-DWI MRI may be most valuable to select patients for surgical resection.

